# Reinforcement omission effects in rats with bilateral lesions in the substantia nigra pars compacta and ventral tegmental area

**DOI:** 10.1590/1414-431X20198303

**Published:** 2019-07-10

**Authors:** T.F. Tavares, D.M. Judice-Daher, J.L.O. Bueno

**Affiliations:** Departamento de Psicologia, Faculdade de Filosofia, Ciências e Letras de Ribeirão Preto, Universidade de São Paulo, Ribeirão Preto, SP, Brasil

**Keywords:** Reinforcement omission effects, Substantia nigra pars compacta, Ventral tegmental area, Fixed interval with a limited hold, Operant behavior

## Abstract

Reinforcement omission effects (ROEs) are characterized by higher response rates after reinforcement omission than after reinforcement delivery. This pattern of behavior is interpreted in terms of motivational and attentional processes. Recent studies from our laboratory have shown that the amygdala, nucleus accumbens, and medial prefrontal cortex are involved in ROE modulation. Also, the literature has demonstrated a role of other areas such as substantia nigra pars compacta (SNc) and the ventral tegmental area (VTA) in processes related to surprising events, such as prediction error and presentation or omission of an event (exteroceptive stimulus and reinforcement). Since these structures send projections to areas related to ROE modulation such as the amygdala, nucleus accumbens, and prefrontal cortex, the objective of the present study was to determine whether the SNc and VTA also integrate the circuit involved in ROE modulation. Rats were trained on a fixed-interval 12 s with limited-hold 6 s signaled schedule of reinforcement (Pre-lesion training). After acquisition of stable performance, the rats received bilateral neurotoxic lesions of the SNc (Experiment 1) and VTA (Experiment 2). Following postoperative recovery, the rats were submitted to two refresher sessions (Post-lesion training). Subsequently, the training was changed from a 100 to a 50% schedule of reinforcement (Post-lesion testing). In both experiments, the results showed that there was no difference in performance between sham rats and rats with bilateral lesions of the SNc or the VTA.

## Introduction

Reinforcement omission effects (ROEs) indicated by greater response strength immediately after omission than after reinforcement delivery have been attributed to both motivational and attentional consequences of the surprising reinforcement omission. For instance, it is reported that the introduction of partial reinforcement in the first goal of a double runway led to a greater response in the second runway immediately after omission than after reinforcement delivery (1 [Bibr B02]). This effect was explained as behavioral facilitation induced by primary frustration (1–3). However, ROEs can also be interpreted in terms of transient behavioral inhibition after reinforcement induced by demotivation or reset of the internal clock (4–6).

Recent studies have shown that the amygdala is involved in the modulation of ROEs. For instance, rats with large amygdala lesions trained to respond under a fixed interval with a limited hold signaled schedule of reinforcement (FI LH signaled) failed to increase response rates during intervals following non-reinforcement (7). However, rats with lesions of the basolateral complex or central nucleus (CeA) of the amygdala trained to respond under the same schedule of reinforcement were more responsive to occasional reinforcement omission than rats of the sham-operated group (8).

The view that amygdala lesions interfere with ROEs is supported by evidence implicating this area to responses correlated with motivational and attentional processes. However, these processes depend on the operation of separate amygdala areas through their connections with other brain systems. Recent studies have investigated whether the ROEs can be modulated by different brain structures linked to the amygdala such as the nucleus accumbens (NAC), medial prefrontal cortex (mPC), and orbitofrontal cortex (OFC). For example, lesions of the NAC and mPC, but not of the OFC, interfered with ROEs in rats submitted to the FI LH signaled schedule (9–11).

Many studies have demonstrated an important role of dopaminergic neurons (DA) in tasks involving the presentation or omission of reinforcement previously associated with exteroceptive stimuli. For example, monkeys presented activation of the DA in the midbrain in the presence of reinforcements and stimuli previously associated with reinforcement (12). The authors suggested that the DA could be involved in transient changes of the basic processes of attention and emotion, underlying learning, and cognitive processes. The activity of the DA when expected reinforcements were omitted was also examined (13).

The ascending pathways of the DA originate from specific areas of the midbrain, such as the substantia nigra pars compacta (SNc) and the ventral tegmental area (VTA) (14,15). Recent studies have assessed the neural activation of these areas in tasks involving both the presentation and the omission of reinforcement, with both procedures being previously associated with exteroceptive stimuli.

The neural activation of VTA and SNc in rats in a procedure involving the prediction error was evaluated (16). The authors examined the role of CeA connections to the SNc and the VTA relative to the unexpected presentation or omission of reinforcement and to the expected presentation or omission of reinforcement, previously associated with a stimulus. Initially, two different retrograde tracers were injected into the SNc and the VTA of rats to label CeA cells. Different groups of rats then received a visual conditioned stimulus (CS) paired or not with food. Finally, Fos induction was assessed after a test session in which rats were exposed to the visual CS alone or paired with food. The results showed that neural activation was greater in projections between the CeA and SNc when the animals received an unexpected omission and delivery of reinforcement than when they received the expected presentation. However, only a small portion of neurons was activated in projections between the CeA and VTA. According to the authors, the interaction between the CeA and the SNc may reflect some attentional or motivational process resulting from surprise due to prediction error. The activation of neural projections from the VTA to CeA in the presence of stimuli associated with reinforcement was demonstrated (17).

Taken together, these data related to structures and circuitry of the SNc and VTA support the idea that these areas seem to be directly involved in associative reinforcement processes.

Thus, the objective of the present study was to determine whether the SNc and VTA also integrate the circuit involved in ROE modulation. Rats were trained in a fixed-interval 12 s with limited-hold 6 s signaled schedule of reinforcement (Pre-lesion training). After acquisition of stable performance, rats received bilateral neurotoxic lesions of the SNc (Experiment 1) and VTA (Experiment 2). Following postoperative recovery, the rats were submitted to two refresher sessions (Post-lesion training). Subsequently, the training was changed from a 100 to a 50% schedule of reinforcement (Post-lesion testing).

## Material and Methods

### Experiment 1

The subjects were 43 experimentally naive male Wistar rats (Central Vivarium, University of São Paulo, Ribeirão Preto Campus, Brazil), 90 days old at the beginning of the experiments, weighing 416 to 433 g. Throughout the experiments, the animals were housed in steel cages in the laboratory colony room on a 12-h light cycle (lights on from 8:00 to 20:00). The rats were maintained on a water deprivation schedule at 85% of their *ad libitum* body weight by limiting access to water. Food was available at all times. The study was approved by the Ethics Committee on Animal Use of the Ribeirão Preto School of Philosophy, Sciences and Literature of the University of São Paulo, Ribeirão Preto Campus, Brazil (protocol No. 13.1.86.53.8).

The animals were anesthetized with an intraperitoneal injection of a mixture containing 0.8 mL of ketamine hydrochloride (0.028 mg/mL) and 0.7 mL of xylazine (3.33 mg/mL). Each rat received 0.1 mL of anesthetic per 100 g body mass. Bilateral neurotoxic lesions of the SNc were made by injecting kainic acid (KA, Sigma, USA; 0.4 mM saline-phosphate buffer, pH 7.4) (18). KA was infused with a 5 µL Hamilton syringe over a 2-min period according to the following coordinates: SNc (n=27): –4.3 mm posterior to bregma and 2.2 mm from the midline, with infusions to a depth of 7.4 mm from the skull surface (0.25 µL per site) (19). The Sham-SNc (n=16) groups received the same surgical treatment, with the exception that no solution was infused (7,8,10,11,20,21). After surgery, all rats received a single subcutaneous injection of 0.1 mL per 100 g body mass (2.15 mg/mL) of Flunixin Meglumine (Banamine®, 50 mg/mL, Intervet, Brazil) for pain relief and were allowed to recover from the surgery for 5–7 days before behavioral testing.

The experiment was conducted in operant chambers (Lafayette model 80201, USA) equipped with a speaker that delivered a 1000-Hz 30-dB tone, a 5-W house-light lamp, and a retractable 5-cm lever. Each chamber was located in a soundproof wooden box provided with a transparent acrylic window held in soundproof experimental rooms. An electrical interface (MRA-Electronic Equipment, Brazil) connected the experimental chambers to a personal computer. This system used a program prepared with Microsoft QuickBasic 4.0 designed for this experiment, which controlled the reinforcement mechanisms and registered and recorded lever presses.

#### Pretraining

Pretraining was carried out over two sessions. In the first session, each rat was trained to press the lever for one 0.05 mL drop of water. The following session consisted of continuous reinforcement training (CRF training). Each session lasted a maximum of 30 min.

#### Pre-lesion training

In the pre-lesion training (15 sessions), rats were trained to respond under a fixed-interval 12 s with limited hold 6 s signaled schedules (FI 12 s LH 6 s). This schedule was presented simultaneously with a tone stimulus of 18 s: the first lever press occurred between 13 and 18 s, resulting in the delivery of 0.05 mL of water. All rats received 20 training trials per session and each trial was interpolated with variable inter-trial intervals (ITI; mean 75 s). At the end of each session, rats were returned to their cages and given sufficient water to maintain their planned body weight schedule. They were deprived of water for approximately 23 h before the beginning of each session.

#### Post-lesion training

After recovery from surgery (approximately 1 week), rats were submitted to two refresher sessions, which were the same as those performed during the pre-lesion training.

#### Post-lesion testing

Rats were submitted to one session (20 trials) in which partial reinforcement was introduced (50% partial reinforcement schedule). Rats were submitted to the same conditions as those of pre-lesion training, but the reinforcement was not delivered after the correct response in half of the trials. Reinforced (10 trials) and non-reinforced trials (10 trials) were randomly distributed during the session using the criteria of up to three subsequent intervals with the same schedule.

After completion of the behavioral training procedures, the rats were sacrificed by asphyxiation with carbon dioxide. Their brains were removed from their skulls, fixed in 10% paraformaldehyde, and then dehydrated in 30% sucrose for cryoprotection (24–48 h). The brains were cut coronally into 40-µm thick sections with a freezing microtome and stained with cresyl violet. Slides were examined under the microscope and neural structures were identified according to Paxinos and Watson's stereotaxic (22).

### Experiment 2

The animal procedures were identical to those of Experiment 1, except that 38 rats were used. The surgical procedures were also identical, except that KA was infused into the VTA with a 5 µL Hamilton syringe over a 2-min period at the following coordinates: VTA (n=25): –4.4 mm posterior to bregma and 0.5 mm from the midline, with infusions at a depth of 7.6 mm (0.2 µL per site) from the skull surface. The Sham-VTA (n=13) group received the same surgical treatment, with the exception that no solution was infused.

The animals underwent the same behavioral training procedures, with the same apparatus.

### Data analyses

The average response of each rat was calculated by dividing the number of bar presses performed by the number of trials. The data of the subjects in each group were grouped to obtain the average bar presses for each period. The response rates were evaluated during the FI 12 s LH 6 s signaled schedule in different phases: pre- and post-lesion training, post-lesion testing after reinforcement (R), and after non-reinforcement (N). For analysis, the FI 12 s LH 6 s schedule was divided into periods of 3 s in the pre- and post-lesion training phases (3 s FI, 6 s FI, 9 s FI, 12 s FI, 3 s LH R, and 6 s LH R) and in the post-lesion test phase (3 s FI, 6 s FI, 9 s FI, 12 s FI, 3 s LH R, 6 s LH R, 3 s LH N, and 6 s LH N).

Data were analyzed by two-way analysis of variance (ANOVA) with group as inter-group factor (SNc and Sham-SNc groups), and period as intra-group factor (3 s FI, 6 s FI, 9 s, FI 12 s, 3 s LH, 6 s LH periods). Significant effects in the ANOVA were followed by the Newman-Keuls *post hoc* test. The level of significance was set at P≤0.05 for all analyses.

## Results of Experiment 1

### Histological results

**Figure 1.**
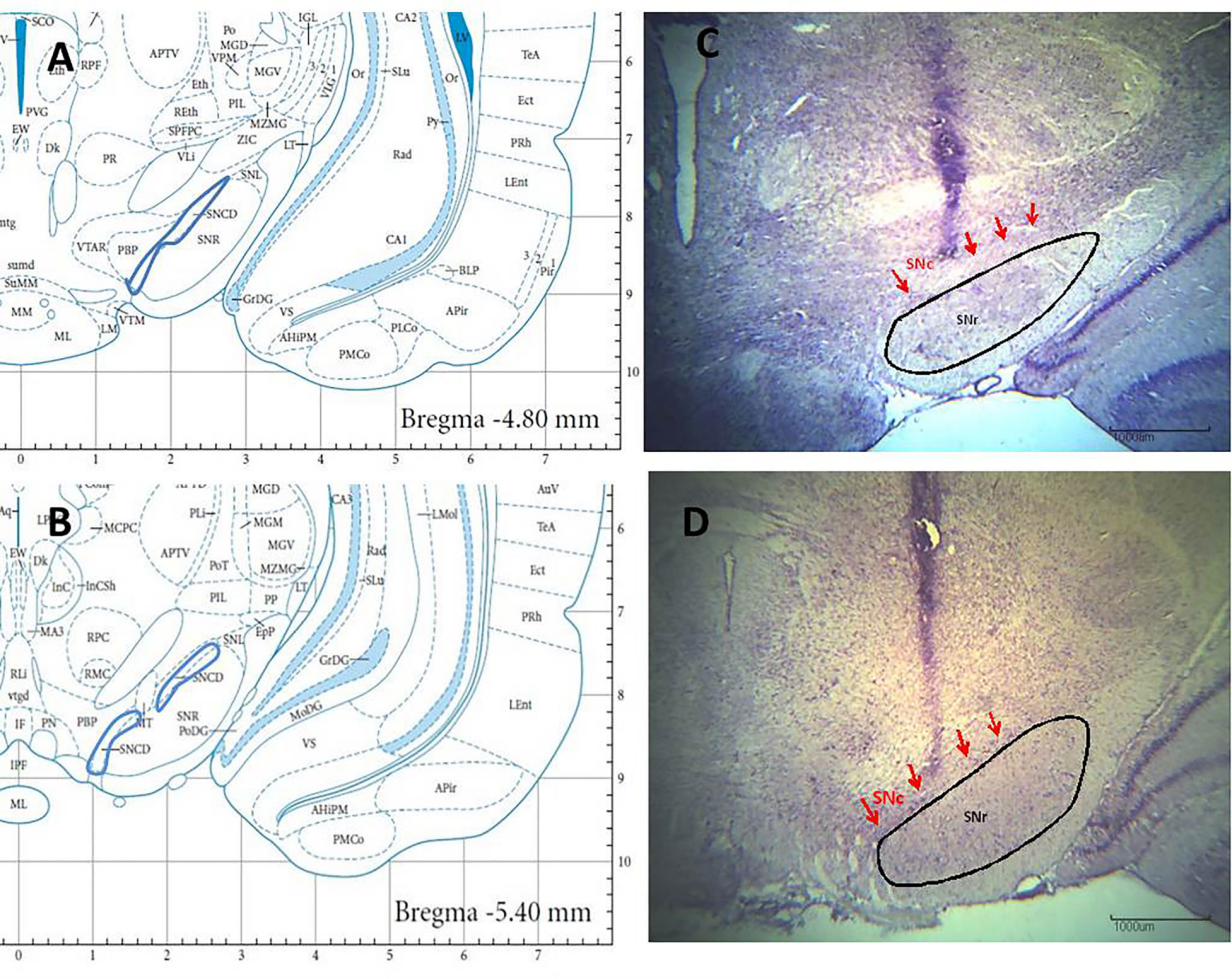
Schematic diagrams (**A**, **B**) and photomicrographs of Nissl-stained cells (**C**, **D**) of the substance pars compacta (SNc) after kainic acid lesion (**C**) or control treatment (**D**). SNr: substantia nigra, reticular part. Magnification 2×, numerical aperture 0.06, wd 7.5; scale bars 1000 μm.

Reliability criteria were established for analysis of the lesions by comparing the borders, extent, and homogeneity of both intact and damaged structures. Only experimental data from the point of the syringe needle correctly located in the substantia nigra pars compacta were used for statistical analysis in the lesioned and sham rats. The point of the syringe needle was positioned symmetrically in the central part of the substantia nigra pars compacta and extended from 4.80 to 5.16 mm posterior to bregma (Figure 1). Ten rats of the SNc group did not survive surgery, and seven rats were discarded because the lesion reached other areas. Data from the remaining rats (n=10) were included in the analysis. Three rats of the SNc-Sham group were discarded because the micro syringe reached other areas. Data from the remaining rats (n=11) were included in the analysis.

### Behavioral results

**Figure 2.**
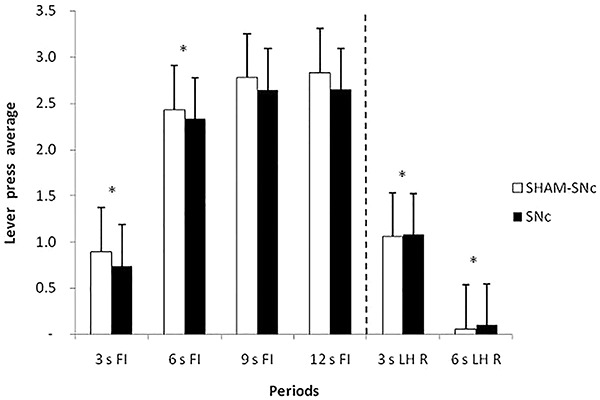
Average response and standard errors in the last three sessions of the pre-lesion training, grouped into 3-s periods, during the periods preceding reinforcement (R) (3 s FI, 6 s FI, 9 s FI, and 12 s FI) and during the periods following R (3 s LH R and 6 s LH R). FI: fixed-interval; LH: limited hold; SNc: substantia nigra pars compacta. *P<0.05 between 3 s FI and 6 s FI; 3 s LH R and 6 s LH R periods for both groups (two-way ANOVA).

ANOVA revealed a significant effect of period (F_5,90_=90.256, P<0.05), but no significant effect between groups (F_1.108_=0.265, P=0.608) and for group × period interaction (F_5,90_=0.640, P=0.670). The Newman-Keuls *post hoc* test showed significant differences between all periods, except between the 9 s FI and 12 s FI periods. These data confirmed task acquisition because of the increased responding during the signaled schedule (FI). The acquisition training data suggest discriminative control during the signal in both the SNc and Sham-SNc groups, which produced different response distributions that depended on timing (Figure 2).

**Figure 3.**
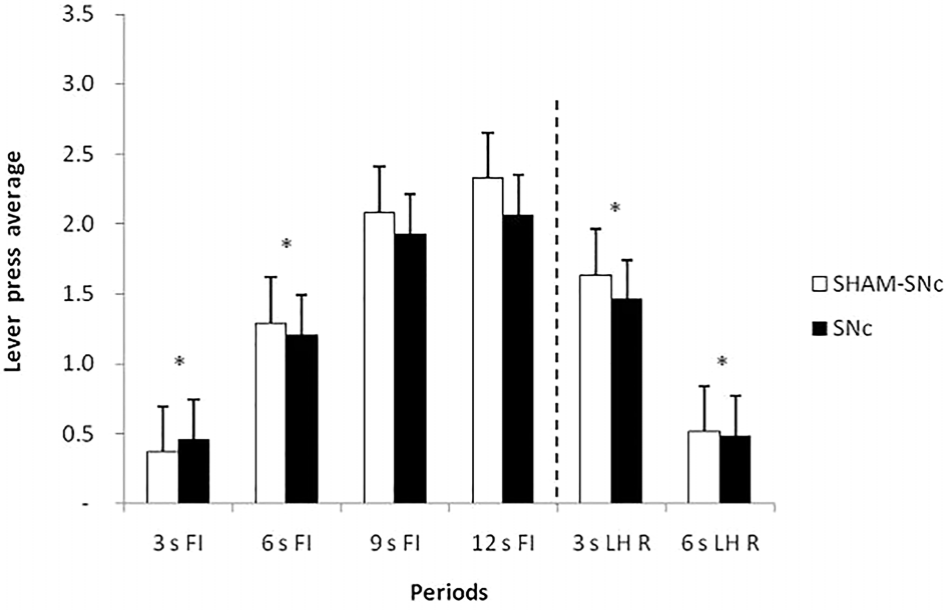
Average response and standard errors of the two sessions of the post-lesion training, grouped into 3-s periods, during the periods preceding reinforcement (R) (3 s FI, 6 s FI, 9 s FI, and 12 s FI) and following R (3 s LH R and 6 s LH R). FI: fixed-interval; LH: limited hold; SNc: substantia nigra pars compacta. *P<0.05 between 3 s FI and 6 s FI; 3 s LH R and 6 s LH R periods for both groups (two-way ANOVA).

A significant effect of period (F_5,90_=85.544, P<0.05), but not between groups (F_1,108_=1.977, P=0.163) and for group × period interaction (F_5,108=_0.673, P=0.645), was found. The Newman-Keuls *post hoc* test showed significant differences between all periods, except between the 9 s FI and 12 s FI periods. These data confirmed that the lesion did not affect task acquisition (Figure 3).

**Figure 4.**
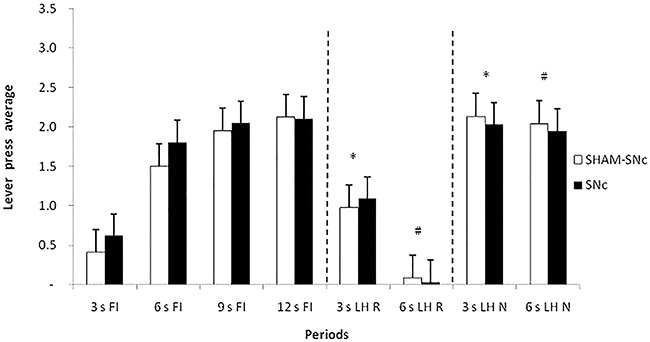
Average response and standard errors in the first session of the post-lesion testing, grouped into 3-s periods, during the periods preceding reinforcement (R) (3 s FI, 6 s FI, 9 s FI, and 12 s FI), following R (3 s LH R and 6 s LH R), and following non-reinforcement (N) (3 s LH N and 6 s LH N). FI: fixed-interval; LH: limited hold; SNc: substantia nigra pars compacta. *^,#^P<0.05 between periods after R and after N (3 s LH R and 3 s LH N; 6 s LH R and 6 s LH N) for both groups (two-way ANOVA).

Figure 4 shows the average response in the first testing session, grouped by 3-s periods, during the periods that preceded the omission or delivery of reinforcement (3 s FI, 6 s FI, 9 s FI, 12 s FI), during the periods that followed delivery reinforcement (R) (3 s LH R, 6 s LH R), and following the omission of reinforcement (N) (3 s LH N, 6 s LH N). ANOVA revealed a significant effect of period (F_8,144_=50.955, P<0.05) but not for group (F_1,162_=2.651, P=0.105) and group × period interaction (F_8,200_=1.72, P=0.724). The Newman-Keuls *post hoc* test showed that the performance of both the SNc and Sham-SNc groups during the periods that followed reinforcement delivery (3 s LH R, 6 s LH R) differed from the response during the periods that followed reinforcement omission (3 s LH N, 6 s LH N). These data indicated that both the SNc and Sham-SNc groups exhibited ROEs. No difference in performance was found between the SNc and Sham-SNc groups during the periods that followed reinforcement omission (3 s LH N and 6 s LH N), suggesting that SNc lesions did not interfere with the ROEs. The *post hoc* test also showed that the period preceding the omission or delivery of reinforcement (12 s FI) did not differ from the periods that followed omission (3 s LH N, 6 s LH N), but differed from the periods that followed reinforcement (3 s LH R, 6 s LH R). Thus, an increase in responding was not observed after non-reinforcement, but a decrease in responding was detected after reinforcement.

## Results of Experiment 2

### Histological results

**Figure 5.**
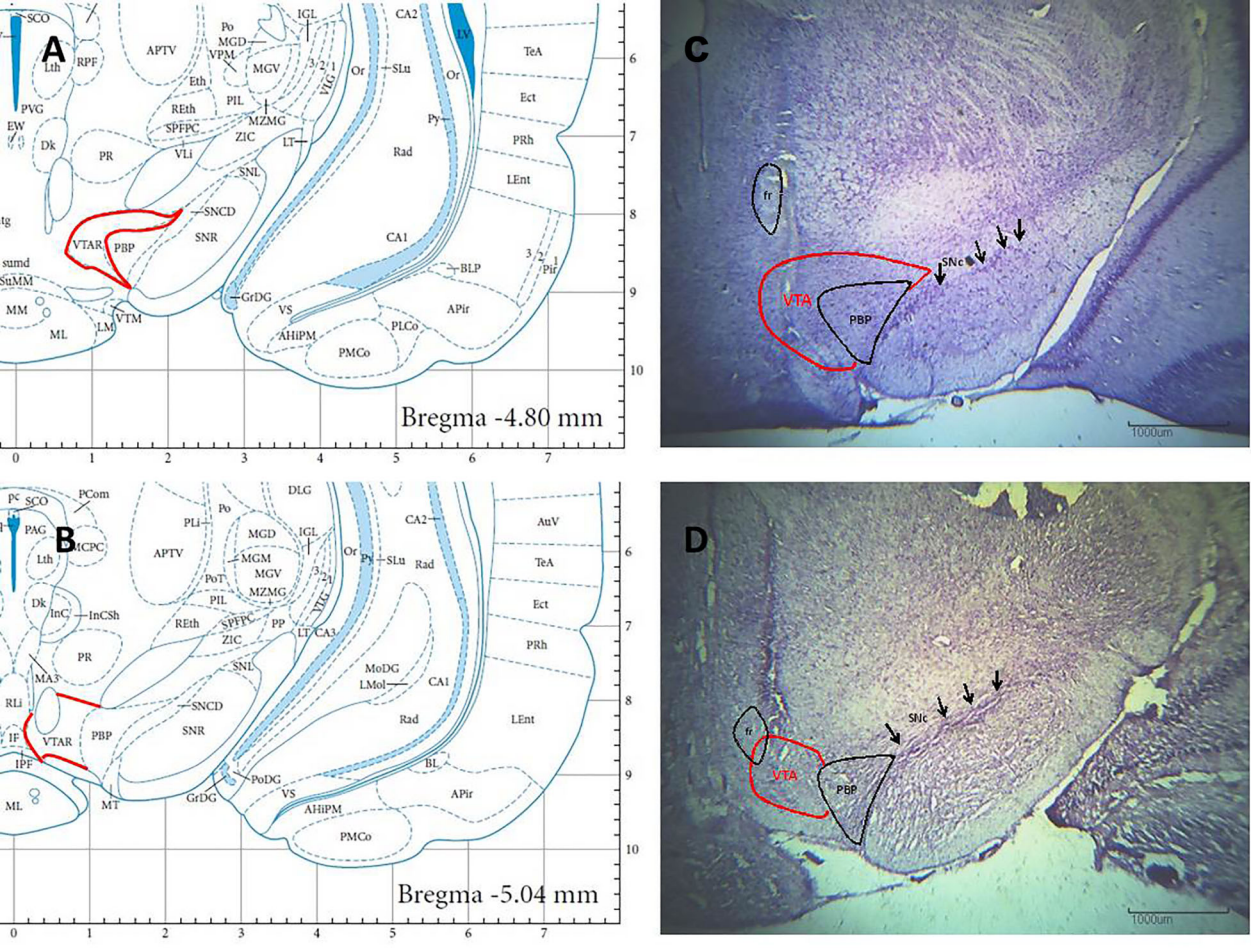
Schematic diagrams (**A**, **B**) and photomicrographs of Nissl-stained cells (**C**, **D**) of the ventral tegmental area (VTA) after kainic acid lesion (**C**) or control treatment (**D**). SNc: substantia nigra pars compacta; PBP: parabrachial pigmented nucleus of the VTA; fr: fasciculus retroflexus. Magnification 2×, numerical aperture 0.06, wd 7.5; scale bars 1000 μm.

In the VTA (Figure 5), the point of the needle was positioned in the middle of the area, extending from 4.80 to 5.04 mm posterior to bregma. Two rats of the VTA group did not survive surgery, and eight rats were discarded because the lesion reached other areas. Data from the remaining rats (n=15) were included in the analysis. Three rats of the VTA-Sham group were discarded because the micro syringe reached other areas. Data from the remaining rats (n=10) were included in the analysis.

### Behavioral results

**Figure 6.**
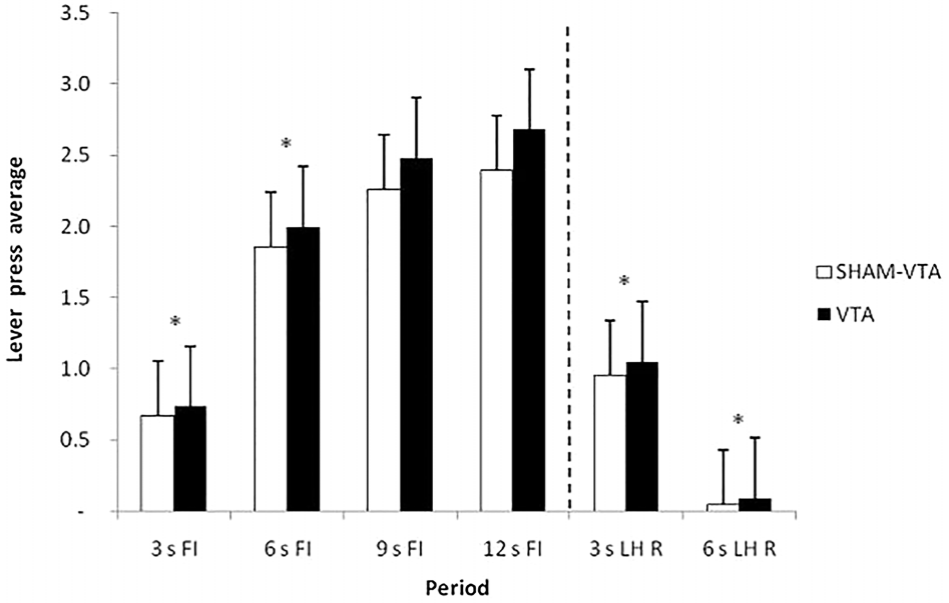
Average response and standard errors in the last three sessions of the pre-lesion training grouped into 3-s periods during the periods preceding reinforcement (R) (3 s FI, 6 s FI, 9 s FI, and 12 s FI) and following (R) (3 s LH R and 6 s LH R). FI: fixed-interval; LH: limited hold; VTA: ventral tegmental area. *P<0.05 between 3 s FI and 6 s FI; 3 s LH R and 6 s LH R periods for both groups (two-way ANOVA).

ANOVA revealed a statistically significant effect of period (F_5,90_=96.535, P<0.001), but not for groups (F_1,90_=0.0908, P=0.767) or group × period interaction (F_5,90_=0.147, P=0.098). The Newman-Keuls *post hoc* test showed significant differences between all periods, except between the 9 s FI and 12 s FI periods for both groups (VTA and Sham-VTA). The acquisition training data suggested a discriminative control during the signal in both the VTA and Sham-VTA groups, which produced different response distributions that depended on timing (Figure 6).

**Figure 7.**
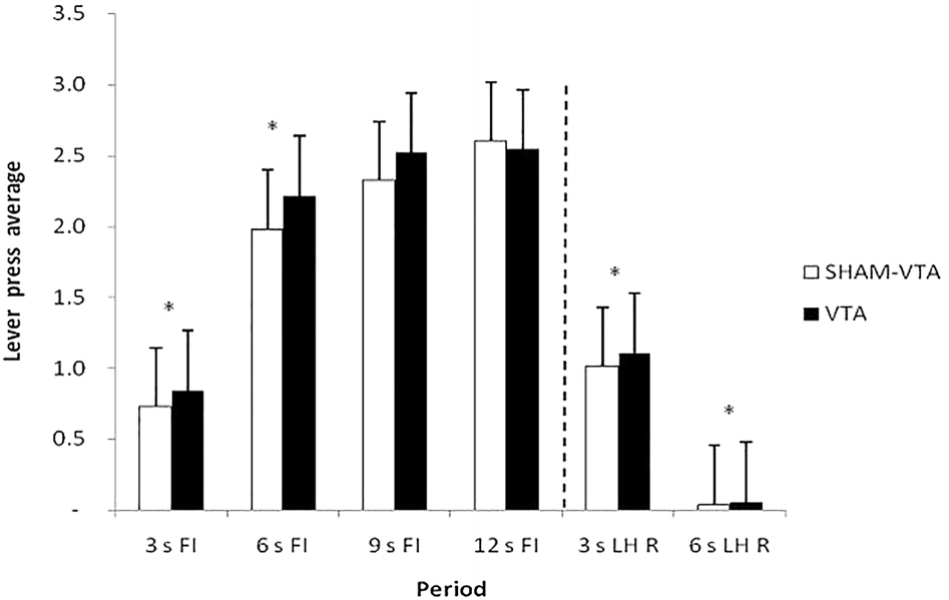
Average response and standard errors in the two sessions of post-lesion training grouped into 3-s periods during the periods preceding reinforcement (R) (3 s FI, 6 s FI, 9 s FI, and 12 s FI) and following R (3 s LH R and 6 s LH R). FI: fixed-interval; LH: limited hold; VTA: ventral tegmental area. *P<0.05 between 3 s FI and 6 s FI; 3 s LH R and 6 s LH R periods for both groups (two-way ANOVA).

A significant effect for period was found (F_5,150_=219.85, P<0.001), but not for groups (F_1,150_=0.380, P=1.0) or group × period interaction (F_5,150_=2.43, P=0.038). The Newman-Keuls *post hoc* test showed significant differences between all periods, except between the 9 s FI and 12 s FI periods. These data confirmed that the lesion did not affect task acquisition (Figure 7).

**Figure 8.**
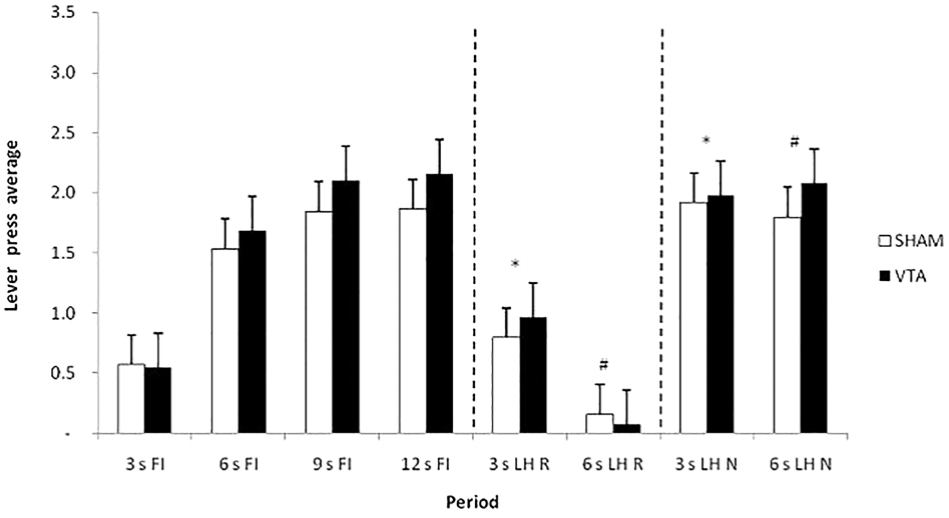
Average response and standard errors in the first session of post-lesion testing, grouped into 3-s periods, during the periods preceding reinforcement (R) (3 s FI, 6 s FI, 9 s FI, and 12 s FI), following R (3 s LH R and 6 s LH R), and following non-reinforcement (N) (3 s LH N and 6 s LH N). FI: fixed-interval; LH: limited hold; VTA: ventral tegmental area. *^,#^P<0.05 between periods after R and after N (3 s LH R and 3 s LH N; 6 s LH R and 6 s LH N) for both groups (two-way ANOVA).

Figure 8 shows the average response in the first test session grouped into 3 s periods, during the periods that preceded the omission or delivery of reinforcement (R) (3 s FI, 6 s FI, 9 s FI, 12 s FI), during the periods that followed R delivery (3 s LH R, 6 s LH R), and following R omission (N) (3 s LH N, 6 s LH N). A significant effect of period was found (F_7,126_=49.397, P<0.001), but not for group (F_1,126_=0.302, P=0.589) or group × period interaction (F_7,126_=0.410, P=0.895). The Newman-Keuls *post hoc* test showed that performance in both the VTA and Sham-VTA groups after R delivery (3 s LH R, 6 s LH R) differed from performance after R omission (3 s LH N, 6 s LH N). These data indicated that both groups exhibited ROEs. No difference in performance was found between the VTA and Sham-VTA groups after R omission (3 s LH N and 6 s LH N), suggesting that VTA lesions did not interfere with the ROEs. The *post hoc* test also showed that the period preceding R omission or delivery (12 s FI) did not differ from the periods that followed the omission (3 s LH N, 6 s LH N), but differed from the periods that followed R (3 s LH R, 6 s LH R). Thus, no response increase was observed without reinforcement, but a decrease was found after reinforcement.

## Discussion

The pre-lesion training data showed a discriminative control during the signal, producing different response rates during FI 12 s LH 6 s signaled schedule for all groups (SNc, Sham-SNc, VTA, and Sham-VTA). The response rates during the initial periods of FI were lower than those observed during the final periods of FI. Furthermore, the response rates during LH showed an opposite distribution. These data characterize the pattern of behavior in FI LH signaled schedules (7,8,10,9,23,24) and can be explained by evoked expectations and the delivery of reinforcement (15,25).

The behavioral pattern displayed by the rats during the last sessions of pre-lesion training was maintained in refresher sessions during post-lesion training. Therefore, the SNc or VTA lesions did not interfere with task acquisition showing that the behavior of the temporal control exercised by the signaled schedule was not affected.

The data of post-lesion testing showed that the Sham groups and lesioned groups (SNc or VTA) presented ROEs: the response rates were higher after reinforcement omission than after reinforcement delivery. Furthermore, there was no difference in response rates between the lesioned groups (SNc or VTA), and those of their respective control groups (Sham-SNc or Sham-VTA). The results also showed that there was no transient behavioral facilitation after non-reinforcement because there was no increase in response rates. On the other hand, there was a transient behavioral inhibition indicated by the suppression of response rates after reinforcement. These same behavioral patterns were observed in other studies (7,9–11).

These results do not agree with studies that evaluated the properties related to reward, prediction error, omission, or presentation of an event (reward or stimulus) (16,17,26). However, in the studies of Lee et al. (26) retrograde trace was used to determine the activation of the SNc and VTA.

The present study was the first to evaluate whether bilateral lesions of the SNc or VTA interfere with properties related to reward such as ROE modulation. However, these structures are very sensitive to manipulation and their disruption can cause several types of damage such as motor and vital events in addition to cognitive damage. This can be verified by the fact that many rats submitted to bilateral lesions presented spasms and became aphagic and adipsic, which led to their death. The animals analyzed in the present study also exhibited spasms after surgery, which decreased during surgical recovery, so that it was possible to subject them to experimental tests.

Thus, these differences may be due to the different procedure used, which can involve different chemical properties and neurotransmitters in the regulation of the SNc and VTA.

Studies evaluating the behavior of rats with bilateral lesion of the SNc or the VTA using KA, as done in the present study, have examined aspects of spatial memory and the impact of dopaminergic lesions on motor ability and motivation (19,27). The effects of VTA or SNc neuron lesion by bilateral KA microinjections have been investigated to clarify the role of the VTA and SNc neurons in learning and memory processes. These lesions significantly decreased spontaneous alternation in the Y-maze task, working memory, and reference memory in the radial 8 arm-maze task in rats, suggesting effects on spatial memory performance. This effect could not be attributed to decreased motor activity because the number of arm entries was not significantly changed (19).

Thus, it seems that KA-induced lesions in the SNc and VTA can disrupt spatial memory, but not aspects involved in ROE modulation. However, further investigations are needed, including a quantification of the lesioned neurons in these areas, to better evaluate these processes since many studies have detected the activation of SNc and VTA when reinforcement was omitted.
